# Supplementary material for: Optimal transport fate mapping resolves T cell differentiation dynamics across tissues
Source: bioRxiv. 2026 Feb 27:2026.02.24.707057. Preprint. [Version 2] doi: 10.64898/2026.02.24.707057 (PMC13160046; doi:10.64898/2026.02.24.707057)
Supplement: Supplement 1 [file NIHPP2026.02.24.707057v2-supplement-1.pdf]

bioRxiv preprint doi: https://doi.org/10.64898/2026.02.24.707057; this version posted February 27, 2026. The copyright holder for this preprint (which was not certified by peer review) is the author/funder, who has granted bioRxiv a license to display the preprint in perpetuity. It is made available under aCC-BY 4.0 International license.

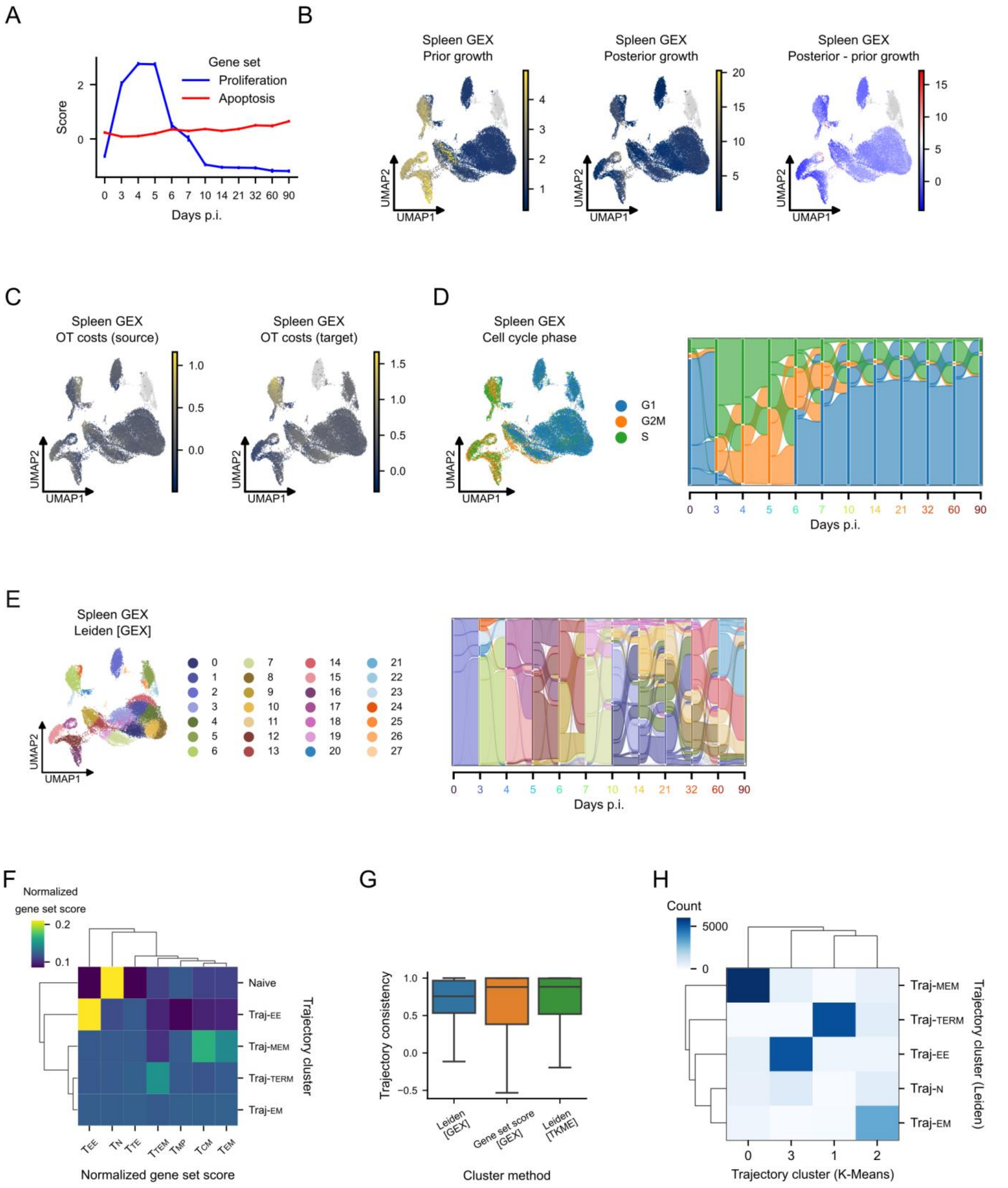

Figure S1: Spleen gene set scores and OT results. **(A)** Mean proliferation (blue) and apoptosis (red) scores over time (error bar = s.e.). **(B)** UMAPs shaded by prior growth rate (left), posterior growth rate (middle), and difference between posterior and prior growth rates (right) used to compute the input OT source marginals corresponding to Figure 1F. **(C)** UMAPs colored by source (left) and target (right) costs of OT trajectory inference in the spleen corresponding to Figure 1F. **(D)** UMAP (left) of estimated cell

bioRxiv preprint doi: https://doi.org/10.64898/2026.02.24.707057; this version posted February 27, 2026. The copyright holder for this preprint (which was not certified by peer review) is the author/funder, who has granted bioRxiv a license to display the preprint in perpetuity. It is made available under aCC-BY 4.0 International license.

cycle phase and Sankey diagram (right) showing probability flow of cells from different phases under OT mapping corresponding to Figure 1F. **(E)** UMAP (left) and Sankey diagram (right) of Leiden clusters based on GEX alone corresponding to Figure 2B,G. **(F)** Heatmap of mean gene set scores per trajectory cluster, with each score associated with a canonical cell state. **(G)** Evaluation of clustering methods on gene expression (GEX) and trajectory embeddings using trajectory consistency metric. Values close to 1 are optimal. **(H)** Confusion matrix displaying the co-occurrence of trajectory clusters using different clustering methods.

bioRxiv preprint doi: https://doi.org/10.64898/2026.02.24.707057; this version posted February 27, 2026. The copyright holder for this preprint (which was not certified by peer review) is the author/funder, who has granted bioRxiv a license to display the preprint in perpetuity. It is made available under aCC-BY 4.0 International license.

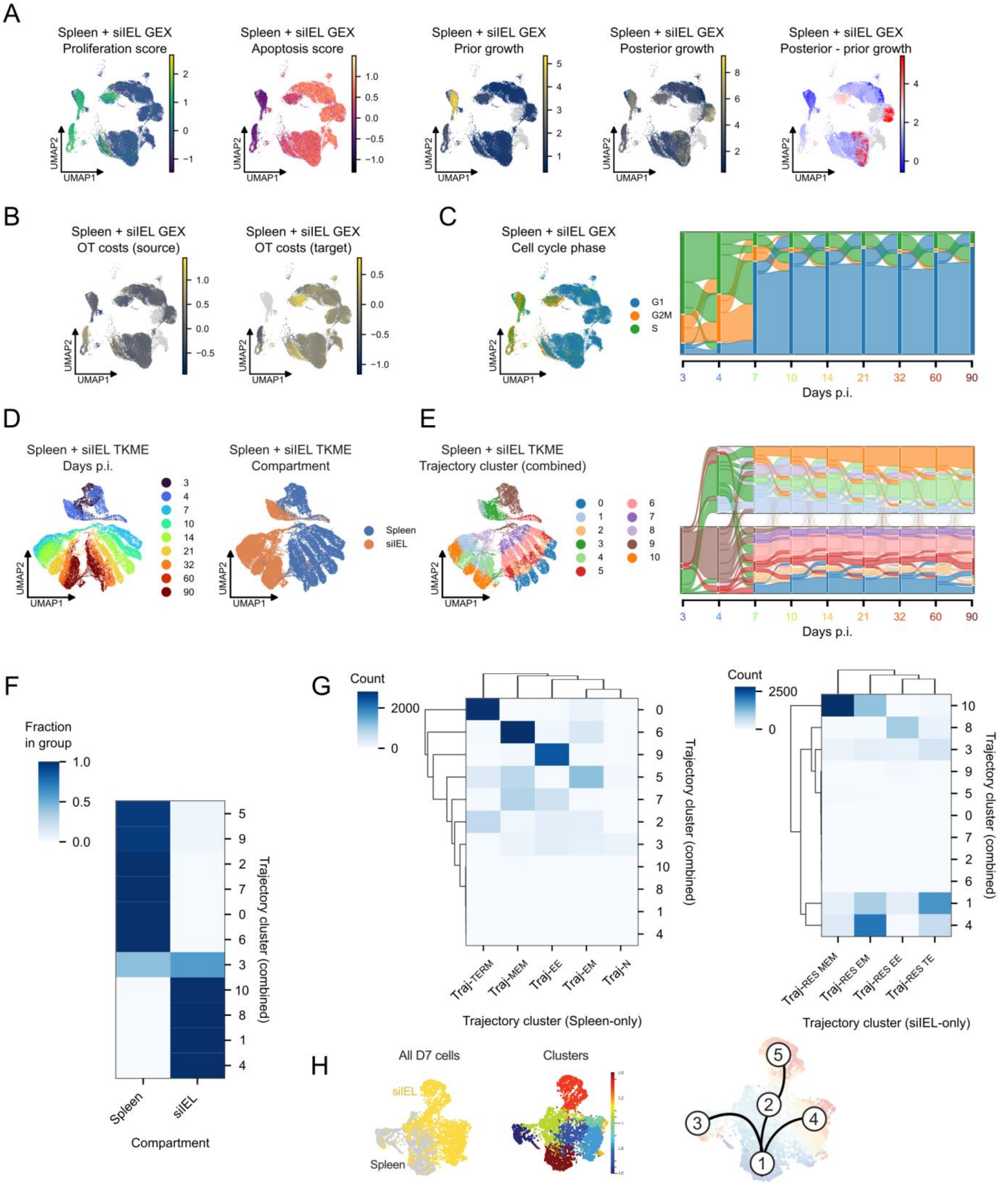

Figure S2: Combined spleen and siIEL gene set scores and OT results. (A) UMAPs displaying quantities related to source marginal estimation corresponding to Figure 3A. From left to right: proliferation and apoptosis gene set scores, initial (prior) and posterior estimates of single cell growth rates, and difference between posterior and prior growth rates. **(B)** UMAPs colored by source and target costs of simultaneous OT trajectory inference for both spleen and siIEL. **(C)** UMAP (left) of estimated cell

bioRxiv preprint doi: https://doi.org/10.64898/2026.02.24.707057; this version posted February 27, 2026. The copyright holder for this preprint (which was not certified by peer review) is the author/funder, who has granted bioRxiv a license to display the preprint in perpetuity. It is made available under aCC-BY 4.0 International license.

cycle phase and Sankey diagram (right) showing probability flow of cells from different phases under OT mapping. **(D)** UMAP dimensionality-reduction based on TKME features derived from the multi-compartment OT model, colored by day (left) and compartment (right) corresponding to Figure 4A. **(E)** TKME UMAP (left) and Sankey diagram (right) displaying clusters derived from multi-compartment TKME features. **(F)** Confusion matrix displaying fraction of trajectory clusters belonging to each compartment corresponding to Figure 4A. **(G)** Confusion matrices displaying overlap sizes between trajectory clusters from the combined multi-compartment model (rows), and trajectory clusters derived from the spleen-specific (left) and siIEL-specific (right) models (corresponding to Figure 4A). **(H)** Leiden clustering of spectral flow cytometry data from Figure 4G.

bioRxiv preprint doi: https://doi.org/10.64898/2026.02.24.707057; this version posted February 27, 2026. The copyright holder for this preprint (which was not certified by peer review) is the author/funder, who has granted bioRxiv a license to display the preprint in perpetuity. It is made available under aCC-BY 4.0 International license.

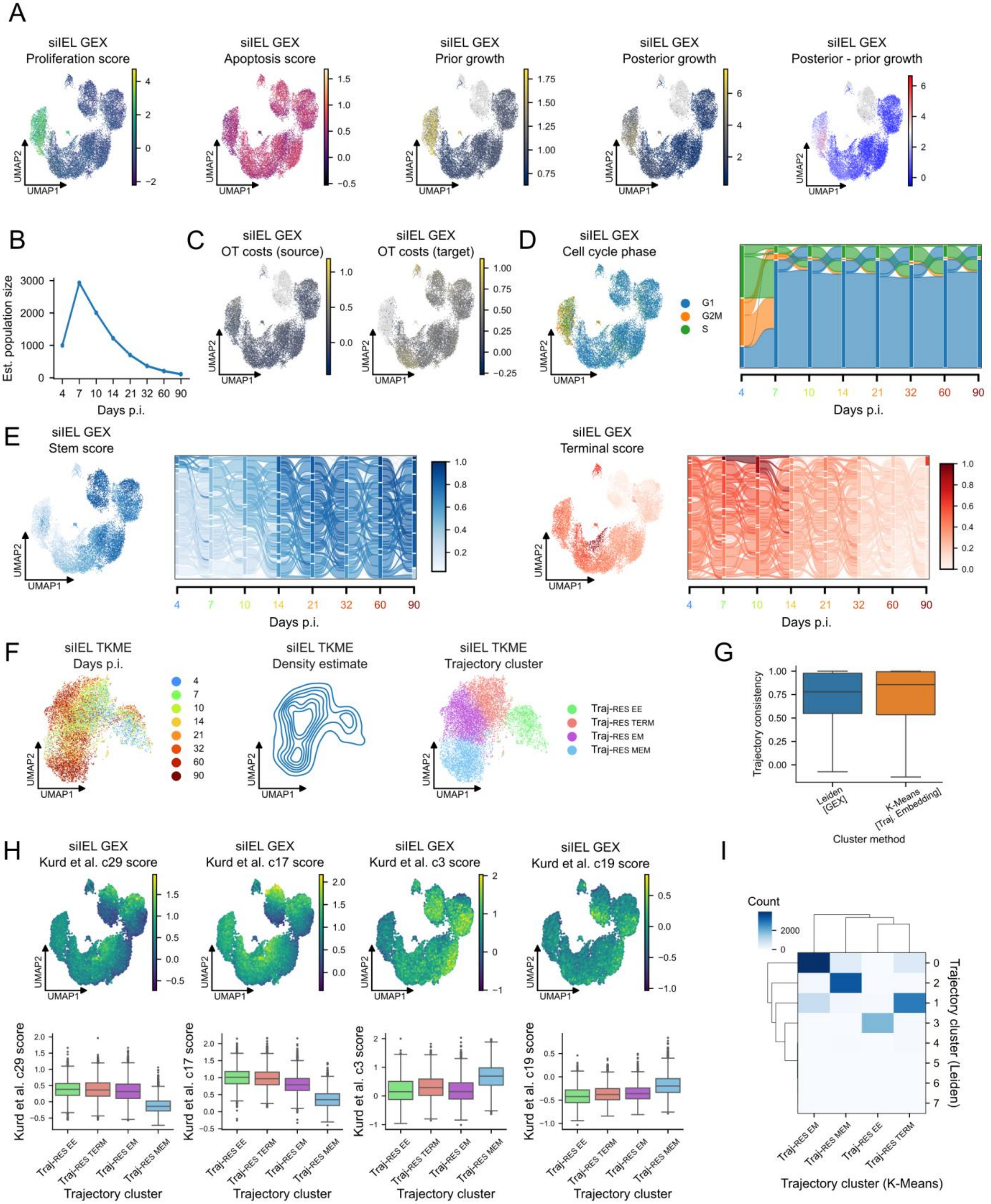

Figure S3: siIEL gene set scores and OT results. (A) UMAPs displaying quantities related to source marginal estimation corresponding to Figure 5A. From left to right: proliferation and apoptosis gene set scores, initial (prior) and posterior estimates of single cell growth rates, and difference between posterior and prior growth rates. **(B)** Estimated siIEL-resident CD8 population size, reconstructed from OT model. **(C)** UMAPs shaded by source (left) and target (right) costs of OT trajectory inference in the siIEL. **(D)**

bioRxiv preprint doi: https://doi.org/10.64898/2026.02.24.707057; this version posted February 27, 2026. The copyright holder for this preprint (which was not certified by peer review) is the author/funder, who has granted bioRxiv a license to display the preprint in perpetuity. It is made available under aCC-BY 4.0 International license.

UMAP (left) of estimated cell cycle phase and Sankey diagram (right) showing fate flow of cells from different phases under OT mapping. **(E)** UMAPs and Sankey diagrams displaying gene set scores for stemness (left) and terminal differentiation (right) corresponding to Figure 5C. **(F)** UMAP dimensionality-reduction based on TKME features derived from the siIEL-specific OT model, shaded by day (left) and trajectory cluster (right), along with kernel density estimate of cell density in the UMAP space (middle) corresponding to Figure 5D. **(G)** Evaluation of clustering methods on gene expression (GEX) and trajectory embeddings using trajectory consistency metric. Values close to 1 are optimal. **(H)** UMAPs (top) displaying gene set scores for TRM cluster signatures reported in (Kurd et al., 2020)). Boxplots (bottom) of median and IQR gene set scores for each trajectory cluster. **(I)** Confusion matrix displaying the overlap size of trajectory clusters using different clustering methods.

bioRxiv preprint doi: https://doi.org/10.64898/2026.02.24.707057; this version posted February 27, 2026. The copyright holder for this preprint (which was not certified by peer review) is the author/funder, who has granted bioRxiv a license to display the preprint in perpetuity. It is made available under aCC-BY 4.0 International license.

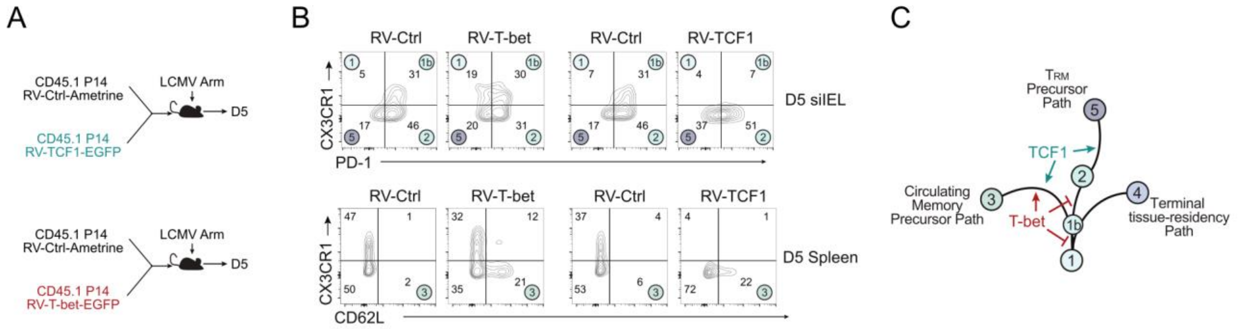

Figure S4: T-bet and TCF1 modulate early circulating and resident differentiation paths. **(A)** Congenic P14 cells transduced with control retrovirus (RV-Ctrl), TCF1-enoding retrovirus (RV-TCF), or T-bet-encoding retrovirus (RV-T-bet) were transferred into mice infected with LCMV Arm and profiled using spectral flow cytometry at D5. **(B)** Flow cytometry analysis of phenotypes in the siIEL (top) and Spleen (bottom). Quadrants are labeled with phenotypes corresponding to SFC clusters from Figure 4M. **(C)** Pseudotime differentiation path from Figure 4M, updated with putative regulatory roles for T-bet and TCF1.
